# Risk and Protective Factors for Executive Function in Vulnerable South African Preschool-Age Children

**DOI:** 10.5334/joc.377

**Published:** 2024-07-18

**Authors:** Caylee J. Cook, Steven J. Howard, Hleliwe Makaula, Rebecca Merkley, Mbulelo Mshudulu, Nosibusiso Tshetu, Gaia Scerif, Catherine E. Draper

**Affiliations:** 1 SAMRC/Wits Developmental Pathways for Health Research Unit, School of Clinical Medicine, Faculty of Health Sciences, University of the Witwatersrand, Johannesburg, South Africa; 2 Early Start and School of Education, University of Wollongong, Wollongong, NSW, Australia; 3 Department of Cognitive Science, Carleton University, Ottawa, Canada; 4 Department of Experimental Psychology, University of Oxford, Oxford, United Kingdom

**Keywords:** Executive function, preschool, contextual factors, Majority World

## Abstract

Executive function (EF) theory and research continues to under-represent the contexts in which the majority of the world’s children reside, despite their potential to support, refute, or refine our current understandings. The current study sought to contribute to our understanding of EF in low-income settings in South Africa by investigating longitudinal associations of context-specific risk and protective factors for EF development in three- to five-year-old children who had limited access to ECCE services before the age of five. Child-caregiver dyads (N = 171) participated in two rounds of data collection (approximately seven months apart) during which child EF was assessed using the Early Years Toolbox; context-specific risk and protective factors were assessed through a caregiver questionnaire. Hierarchical linear regressions revealed that after controlling for age, attending ECCE services at time 2 (*β = 0.30, p < 0.001*), and diversity of caregivers at time 1 (*β = 0.14, p = 0.041*) were the only factors positively associated with EF at time 2. Other factors commonly associated with EF such as caregiver education, and household income were not significant, while resources in the home were significantly associated with EF (*β = –0.18, p = 0.007*) but in the opposite direction to what was expected. These results add to accumulating evidence that predictors of EF established in Minority World contexts may not be consistent across contexts, emphasising the need to broaden the EF evidence base. For instance, future studies could incorporate qualitative and ethnographic methods to better capture the cultural and contextual nuances relating to EF, to better inform our statistical and theoretical models.

## Introduction

Executive functions (EFs) are a set of complex, higher-order cognitive skills that emerge in early childhood and are used throughout our lifespan. EFs help children to: hold and work with information in mind (working memory); resist distractions and maladaptive impulses (inhibition); and flexibly reorient attention as situations require (cognitive flexibility) (Miyake & Friedman, 2012). EF has garnered a lot of attention over the years due to its prediction of broad later-life outcomes such as academic achievement, healthy lifestyles, substance abuse, and quality of life (Fuhs et al., 2014; Moffitt et al., 2011). As is commonly the case in Psychology (Nielsen et al., 2017), most of what we know about EF, including the definition and conceptualisation derives from Minority World countries (Haslam et al., 2019), elsewhere also referred to as high-income, or ‘W.E.I.R.D.’ (Western, Educated, Industrialized, Rich, Democratic, Henrich et al., 2010). There is an increasing preference for reference to Majority and Minority World countries (Alam, 2019; Khan et al., 2022) as they better highlight the under-representation of most children and their contexts in child development research (Draper et al., 2022). There has been an increase in EF studies from Majority World settings in recent years, with a focus on low-income and low-resource contexts (Cook et al., 2023; Mousavi & Gharibzadeh, 2021; Nweze et al., 2021; Suntheimer et al., 2022; Willoughby et al., 2019; Wolf & McCoy, 2019). The evidence available thus far has highlighted similarities with research from Minority World Countries in terms of criterion validity, biological basis, and associations with broader outcomes suggesting EF skills may be culturally universal (Obradović & Willoughby, 2019). In contrast, predictors of EF in early childhood in Majority World countries have shown some deviation from Minority World Countries. For example, a study in Nigeria found that early adversity resulted in enhanced working memory (Nweze et al., 2021), contrary to the widely believed notion that early adversity impairs cognition. Children use their EF to achieve their own specific goals, which are influenced by the context in which they are developing (Doebel, 2020), and researchers have recently focused on understanding how the development of EF is expressed in diverse contexts (Haslam et al., 2019). Yet, representing Majority World settings remains a challenge in terms of proportionate publication and the attention they attract, to meaningfully influence prevailing theories of EF and its development (Draper et al., 2022).

### Currently known predictors of executive function

EF researchers have spent considerable effort identifying the antecedents of EF, in hope of identifying the mechanisms that can realise the extensive benefits implied by longitudinal associations of EF with broad later-life outcomes (e.g., Ahmed et al., 2018; Moffitt et al., 2011). One prominent but less malleable antecedent (at least directly) is household socioeconomic status (SES). The SES gradient of EF (low SES is associated with lower EF) has been replicated across multiple Minority World contexts (e.g., Canada: Vrantsidis et al., 2020; United States: Hackman et al., 2015; United Kingdom: Blakey et al., 2020). This implies the hypothesis that children from low-income settings in low- and middle-income countries are at greater risk for poor EF development compared to their higher income country counterparts. However, recent cross-cultural research suggests that some children from low- and middle-income countries, even from the lowest-SES groups in those contexts, perform comparably well on EF tasks as children in high-income countries (Howard et al., 2020; Metaferia et al., 2021). Additionally EF has been found to mediate the relationship between SES and academic achievement (Haft & Hoeft, 2017; Lawson & Farah, 2017; Raver et al., 2013). This suggests a need to explore the complex interplay of risks and protective factors for EF development in low-income settings in Majority World contexts. Such findings might have implications for prevailing theories of EF development more specifically, and cognitive development more broadly, which presently appear unable to explain some of these ‘counter-intuitive’ findings.

Home and community factors that have been identified as predictors in EF research in Minority World countries routinely implicate: parenting practices; number of siblings; home learning environment; access to high quality early childhood care and education (ECCE); and exposure to stressors such as violence. (Blair et al., 2014; Cole & Mitchell, 2000; Hughes & Devine, 2019; McAlister & Peterson, 2006; Sasser et al., 2017). These factors are conflated in associations of EF with the home learning environment, which is often assessed in terms of the quantity and quality of a parent’s engagement in enrichment activities with their child (Snelling & Dawes, 2010). Yet research linking the home learning environment to EF has yielded mixed results, suggesting complexity in how home-based factors related to EF growth. For example, interventions aimed at increasing parental support and enrichment have been found to improve children’s EF skills (Obradović et al., 2017), yet other studies report null associations between stimulation in the home and child EF skills (Lohndorf et al., 2021; Röthlisberger et al., 2010; Zysset et al., 2018).

These home and community factors have typically been measured and studied based on Western norms, in which the focus tends to be on one-on-one parent-child interactions (typically the mother), and nuclear family households. This is not representative of the home environment for many children in Majority World countries, particularly those in low-income settings. Instead, many children live in multi-generation households and interact with a broad range of caregivers and social partners (Bradley & Corwyn, 2005; Morelli et al., 2018). It is thus not unexpected that, while some findings on home and community factors from Minority World countries align with those from Majority World countries, others do not. For example, aligning with Minority World findings, a study in Cote d’Ivoire found that higher levels of enrichment in the home (help with academic activities) was associated with better EF task performance, even though the home environment included non-nuclear family characteristics such as a lower number of literate adults in the household (Jasińska et al., 2022). Similarly, a study with 6-year-old children in Zambia revealed that reading activities at home accounted for the association between SES and EF (McCoy, Zuilkowski, et al., 2015), as is also found in Minority World countries (e.g., Hamilton et al., 2016). Furthermore, a recent study in young children from Pakistan revealed that having a higher number of older siblings was associated with better EF performance (Rathore et al., 2022) Yet not all findings have been consistent with Minority World theorising and evidence. For instance, a recent study in The Gambia found a significant positive association between the number of *caregivers* and EF performance (Milosavljevic et al., 2023). In Chile, supportive discipline (maternal sensitivity with positive discipline), but not parental cognitive stimulation, predicted school readiness – while neither predicted EF (Lohndorf et al., 2021).

Another commonly found home and community predictor of EF in early childhood is stress. Population studies show that children and families in low-income settings in both Minority and Majority World Countries are exposed to disproportionately higher amounts of stressors (e.g., economic hardship, chaotic environments, etc.) compared to higher income families (Duran et al., 2020; Evans & Kim, 2013). Exposure to violence, in particular, appears to be negatively associated with child EF and self-regulation (McCoy, 2013; McCoy et al., 2016; McCoy, Raver, et al., 2015). Although the mechanisms of this detrimental effect are still debated, multiple pathways are plausible, including hypothalamic pituitary-adrenal axis functioning, cortisol release and neural connectivity (Blair, 2010; Ganzel et al., 2010), and some have even argued that EF may be used as an adaptive skill (Nweze et al., 2021). A recent study in South Africa preschool children did not find associations between exposure to violence and child self-regulation (Cook et al., 2022).

Regardless of the reasons, this pattern of divergent evidence suggests the presence of risk and protective factors in Majority World family contexts that do not clearly conform to findings and expectations from Minority World contexts. The mixed nature of the limited EF evidence from Majority World countries may in part be a result of different cultural norms and how they affect the manifestation and meaning of certain practices and occurrences (Halgunseth et al., 2006).

### South African context

South Africa is considered a Majority World country, the most unequal in the world, with pervasive poverty and inequity. A recent national study highlighted the impact of this inequity on early childhood development, reporting that 65% of four-five-year-old South Africa children attending ECCE services face barriers to thriving, with only 41% on track for cognitive development based on South African norms (Tredoux et al., 2024). The detrimental effect of poverty in South Africa is further illustrated by the school achievement gap between high- and low-income children (Dawes et al., 2020; Draper et al., 2012; Lessing & De Witte, 2005; Naudé et al., 2003; Save the Children, 2016).

Currently, all ECCE services for children aged three-five years in South Africa are privately run and require fees for attendance, creating a barrier to enrolment for low-income children. However, in the year that children turn six, they can start Reception year (Grade R) in a ‘no-fee’ government school, but there are issues with the quality of education provided and challenges with over-crowded classrooms (Richter & Samuels, 2018).

Qualitative findings from caregivers of young children not attending ECCE services in vulnerable settings (Draper et al., 2023) have already begun to contextualise identified risk and protective factors for child development. Risk factors mentioned by caregivers included those previously identified: low-socioeconomic status, dysfunctional family relationships, caregiver mental health, violence and crime. Yet the caregivers downplayed their perceived impact on their child. For example, some commented that violence and crime is the norm and children may be desensitised to it. Protective factors mentioned by the caregivers included social support from family and community members, access to early learning resources, and infrastructure in the community such as parks and libraries. In terms of their own role as a caregiver, they emphasized developmental activities they do with their child, as well as their aspirations for access to more stimulating and beneficial activities (e.g., sports, visiting the aquarium). However, complementing these qualitative insights with a quantitative investigation of the modifiable risk and protective factors of EF in this context could help inform not only more appropriate local EF interventions, but could also provide insights that better elaborate and nuanced global theories about EF development. Although some previous studies have investigated the cross-sectional associations of known EF predictors (Cook et al., 2019; Milosavljevic et al., 2023), there have been no longitudinal investigations undertaken and none looking specifically at children who do not have access to ECCE. This is an important, yet understudied and uniquely vulnerable group, who constitute a significant proportion (30%) of the population of South African three-five-year-olds (Hall et al., 2019). The impact of household and community influences on EF may be even more pronounced in this group, given they are spending all their time at home and in their communities (Draper et al., 2023).

### The current study

The current study seeks to contribute to our understanding of EF in low-income settings in South Africa by illuminating these contextual and cultural nuances. This study builds on previous cross-sectional research, and aimed to investigate the longitudinal associations of context-specific risk (e.g., exposure to violence, negative parenting practices, low caregiver education) and protective factors (e.g., positive parenting, enrichment activities in the home, siblings/other children in the home) for EF development in three- to five-year-old children from low-income settings in South Africa who cannot typically access ECCE. This was done by looking at longitudinal predictors of EF (aim 1) and predictors of EF change (aim 2).

We hypothesized that positive caregiver-child relationships, family relationships, >1 siblings/children in the home, and quality of home learning environment (e.g., number of home learning activities, toys and books in the home) would predict better EF. In addition to these predictors the current study was also able to investigate whether starting ECCE services between the two rounds of data collection predicted EF at time two.

## Methods

### Study design

**Figure 1:**
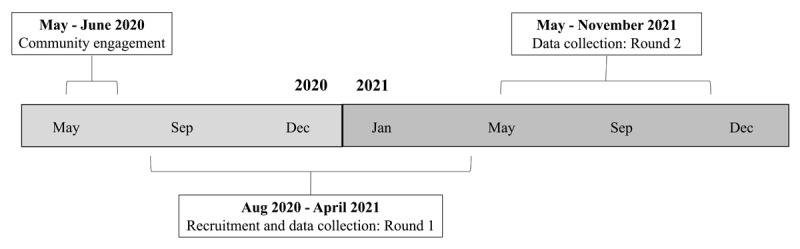
Data collection timeline.

The data reported in this paper derive from a larger longitudinal study aiming to understand the barriers and potential of ECCE in low-income South Africa settings. Other data from the larger study have been published elsewhere, focussing on cross-sectional associations of self-regulation and exposure to violence, numeracy and the home learning environment, EF with the home learning environment and family characteristics (Cook et al., 2022; Merkley et al., 2023; Milosavljevic et al., 2023), as well as qualitative insights on child development (Draper et al., 2022). Previous multi-national investigations have only considered data from Time 1 in the current dataset, whereas the current study includes data from two timepoints (in 2020 and 2021, approximately seven months apart; Figure 1) and considers all collected variables for analysis. More specifically, for the current analysis, predictors of EF were captured at time 1 while EF was captured at both time 1 and time 2 to capture change in EF. The only predictor variables taken from time 2 were the child’s age (control variable) and whether the child had started ECCE. Table 1 reports the full list of measures included in the larger study, as well as whether they were collected at time 1, time 2, or at both time points (an asterisk * denotes measures included in the current study). A detailed timeline of the COVID-19 pandemic and restrictions in relation to the study timeline is provided as supplementary material.

**Table 1:** Timing of data collection measures.


MEASURE	TIME 1	TIME 2

*Caregiver reported*	✔	✔

Demographic details *	✔	✔

Child attending ECCE services *		✔

Socioeconomic status *	✔	✔

Home Learning Environment *	✔	✔

Parenting and Family Adjustment Scales *	✔	✔

Child Exposure to Community Violence checklist *	✔	✔

COVID-19 pandemic related questions	✔	✔

*Child direct assessment*		

Executive function – Early Years Toolbox *	✔	✔

Early numeracy – Give N	✔	✔

School readiness – International Development and Early Learning Assessment		✔


*Note:* ECCE = Early childhood care and education.

### Study sites

Participants were recruited from four diverse low-income communities within the Cape Town Metropolitan area. Two of the communities consist of mostly informal housing or dwellings, which are defined as a makeshift structure, not approved by the local authority, and not meant for permanent residence. Overcrowding is an issue in these communities (population density 16,957.67 per km^2^ and 10,120.31 per km^2^); other challenges include high rates of unemployment (21% in the Western Cape), food insecurity, alcohol abuse, crime and HIV (Statistics South Africa, 2011). The other two communities consist of both formal and informal housing. The population density varies throughout these communities (between 4255.94 per km^2^ and 16553.99 per km^2^). Gang activity and drug abuse are major challenges in these communities, in addition to high rates of unemployment (statistics as above), crime, and food insecurity (Statistics South Africa, 2011).

### Participants

This study recruited 243 children aged three to five years (M_age_ = 4 years, 8 months; 51.9% female) and their primary caregiver from these low-income settings. Specifically, children who were not attending ECCE services were recruited however, at the second round of data collection, just over half of the caregivers reported that their child had started attending ECCE. This is likely because the second round of data collection occurred in a new academic year meaning that children who were five- turning six-years-old were able to start their reception year at school (still considered ECCE as explained earlier), or that family circumstances had changed to allow the child to attend ECCE services. Although the study set out to recruit children who were not accessing ECCE services, the delay in the start of the study caused by the COVID-19 pandemic meant that the second round of data collection fell in a new academic year. Therefore, many of the child participants who were turning six years old in that year were able to start their Reception year. Additionally, it was possible that changes in family circumstances in the new year could result in the child participant being able to attend an ECCE service. Therefore, it was crucial that this transition was captured and explored as a possible predictor of EF.

Recruitment of children and a primary adult caregiver (>18 years) was facilitated through a community-based organisation who implement a home-based stimulation programme for preschool age children and their families living in environments characterised by poverty, unemployment, crime, and violence. Contact numbers of interested caregivers were passed on by the organisation’s home visit staff to research assistants. Due to COVID-19 restrictions in place at the time, initial recruitment took place telephonically. As such, the research time called potential participants to provide information about the study, answer any questions, invite caregivers to participate, and (once meeting in-person was possible) obtain written informed consent.

From the initially recruited sample of 243, 60 dyads could not be included in the final analyses. Reasons for insufficient data capture for current analyses were: 11% lost due to inability to reach the caregiver and child in person at time 1 (due to COVID-19 lockdowns, some of these needed to be pursued telephonically) and a further 22% at time two. Reasons for this loss to follow up varied, including change of phone number, losing their phone, or moving out of the community. Participants lost to follow up did not differ significantly in child age, caregiver age, caregiver education, household assets, exposure to violence, or the home learning environment. Demographic data that was missing at Time 1 (including child age, child sex, and caregiver education) but available at Time 2 was inferred to maximise the available data. Additional missing data was very low (<2%) and therefore no imputation on available data was done.

### Measures

#### Caregiver Questionnaire

The caregiver questionnaire included demographic, socioeconomic and household details, as well as questions to assess child exposure to community violence, parent and family adjustment, and home learning environment. Caregivers were asked to provide their child’s age and sex, and to indicate if they were attending ECCE services (this could have included their reception year, Grade R, which children can attend in the year they turn 6 years old). Although the study sought to recruit children who were not accessing ECCE, it was important to include this variable to capture any changes between the two rounds of data collection that may have occurred as a result of the new academic year or a change in circumstances for the participants.

*Caregiver education* level was obtained by asking the caregiver how many years of school they completed, whether they completed any post school education, and their highest qualification. Total caregiver education was calculated by summing the total number of years in school and the number associated with the type of post school education (1 for certificate, 2 for diploma, 3 for degree).

To assess *socioeconomic status*, selected items (household assets and income range) were replicated from the National Income Dynamics Survey used nationally across South Africa (http://www.nids.uct.ac.za). The household assets survey has 26 items (e.g., television, lounge set, cell phone, fridge), and caregivers are asked to report whether their household has these items. The household assets score is a sum of the items present in the household. The income range is determined by asking the participant’s household income is above, the same as, or less than a certain range.

*Child Exposure to Community Violence Checklist* (CECV; Martin et al., 2013) was used to assess the child’s exposure to violence in the past year. The 29-item CECV initially described by Martin and colleagues (2013) has been used in other South Africa studies of preschool-age children from low-income settings (Cook et al., 2022; Donald et al., 2018) This contains 29 questions about whether a child has witnessed or experienced general violent/criminal acts, family and non-family violence, and or had feelings/experiences of not being safe. Questions are answered on the following scale: never; once; twice; three to ten times; more than ten times. This scale yielded a total score with a higher score indicating higher volume of exposure to community violence. The total CECV showed acceptable internal consistency (alpha = 0.72).

*Parenting and Family Adjustment Scales* (PAFAS; Sanders et al., 2014) is a 30-item inventory to assess parenting practice and family adjustment. It has been used in South Africa with a similar sample of child and caregiver pairs from low-income settings (Donald et al., 2018). Each item, or parenting behaviour (e.g., I praise my child when they behave well), is rated on a 4-point scale from 0 (not true at all) to 3 (very much true), referring to the past 4 weeks. Scores can be summed to yield a total score, as well as subscale scores for: parental consistency, coercive parenting, positive encouragement, caregiver-child relationship, family relationships and parental adjustment. For each subscale, items were summed such that a higher score indicates higher levels of dysfunction. Only caregiver-child relationship (α = 0.83) and family relationships (α = 0.67) showed acceptable internal consistency, and were included in the analysis.

*The Home Learning Environment* questionnaire (Dawes et al., 2020) was used to assess factors in the home that influence learning. This questionnaire was developed for use in South Africa by combining items from the UNICEF Multiple Indicator Cluster Survey (https://mics.unicef.org) and the Home Learning Environment questionnaire (Melhuish, 2010), which were further adapted to ensure relevance to the local context. The resultant scale evaluates the quality of the home environment by asking yes or no questions about the variety of toys and books available in the home, asking how frequently (never, sometimes or many times) the child engaged in eight enrichment activities over the last 7 days, the caregivers that did the activities with the child (e.g., reading books, counting things), and asking how much time (very little, some time, lots of time) the caregiver has available to spend with their child. The four variables derived from these questions include (1) the home learning activity (HLA) frequency (i.e., sum of the different activities the child did with a higher score indicating a higher frequency), (2) the diversity of caregivers (number of unique caregivers that did HLA activities with the child), (3) the total books and toys in the house (sum of the yes answers to having a variety of toys and books in the house), and (4) the total time available to spend with child.

#### Executive function

EF was assessed using EF measures of the Early Years Toolbox (EYT; Howard & Melhuish, 2017) suite of iPad-based assessments, namely: Go/No-Go (inhibition), Card Sort (cognitive flexibility) and Mr Ant (working memory). EYT tasks had integrated audio instructions in local languages to ensure consistent delivery, sequencing and timing. These measures have been successfully used in previous studies within South Africa (Cook et al., 2019; Draper et al., 2020) and other Majority World countries (e.g., Bangladesh: Hossain et al., 2021, Zimbabwe: Munambah et al., 2021). *Go/No-Go* asks children to respond to ‘go’ trials (catch a fish by tapping the screen, presented for 80% of trials) and refrain from responding to ‘no-go’ trials (avoid the sharks by resisting tapping the screen). Inhibition was indexed by an impulse control score that represents the product of the Go and No-Go proportional accuracy (per protocols of Howard & Melhuish, 2017). *Card Sorting* asks children to sort stimuli (i.e., blue rabbits, red boats) first by one dimension (e.g., colour), then by the other dimension (e.g., shape). There is a third dimension where stimuli must be sorted by colour if it is surrounded by a black border or, if there is no black border, stimuli are sorted by shape. Cognitive flexibility was indexed by the number of correct sorts that occurred after the initial pre-switch trials. *Mr Ant* asks participants to remember the location of an increasing number of stickers on a cartoon ant. The task proceeds as follows: Mr Ant appears with one or more stickers for 5 s; then a blank screen is presented for 5 s; finally, Mr Ant reappears without any stickers and children tap the locations they believe the stickers were. Working memory is indexed by a point score that estimates working memory capacity (per protocols of Howard & Melhuish, 2017). While the structure of EF in Majority World settings and if/how this might differ across development is not yet settled–and our study was not designed to determine this–our interest and hypotheses for this early study of risk and protective factors concerned development of EF more generally (rather than having hypotheses for each EF component). This is compatible with both one- and multiple-component accounts of EF, as even multi-component models of EF find robust common variance between factors (whether or not optimal fit is produced by a one-factor EF model). In creating our EF variable, (Camerota et al., 2020) advocate for modelling this as a composite (rather than latent) variable – given genuine and consequential questions on what EF latent variables capture (and the extent to which this is truly a ‘purer’ index of EF that gives rise to manifest performance on constituent EF indices that comprise the latent variable). Accordingly, we adopted a composite approach in line with their findings and recommendations.

### Procedure

This study was approved by the Ethics Committee of the University of Witwatersrand (M200104). Written informed consent was provided by adult participants, and parent/caregiver consent for children. All testing was conducted by trained research assistants who could speak the home language of participants (isiXhosa, English or Afrikaans in the current study) and instructions for the EF tasks were translated into the appropriate home language. Due to COVID-19 restrictions and that some recruitment areas posed a safety risk to research assistants, testing took place in various ways. During South Africa’s lockdown, 30 caregiver questionnaires were done telephonically. As child testing needed to be done in person, this occurred once lockdown restrictions eased. For these 30 participants, the time between caregiver questionnaire and child testing ranged from five days to 95 days (M = 50 days). The remainder of the questionnaires were administered in person by research assistants and on the same day as the child tests. For the two areas that posed a safety risk for the research assistants, testing was done at a central community venue. The children participated in the tasks individually with guidance from the research assistant. Incentives for participation were provided, as well as transport reimbursement where necessary.

### Data analysis

Data were analysed using Stata 17 (StataCorp. 2021). Exploration of the data indicated that assumptions of planned analyses (i.e., normality, collinearity, etc.) were met. All variables were checked for outliers, and only four variables had extreme outliers. It was decided that trimming outliers was not appropriate for this study given the smaller sample size, as it would result in a loss of information, reduced statistical power, and biased parameter estimates. Given the high number of potential independent variables, bivariate correlations (see table S1 in supplementary material) and local polynomial regressions were used to determine independent variables that were important for explaining the variance in the dependent variable. Thereafter, variables deemed important from these analyses, as well as variables that are considered important control variables in this field (child age, child sex) were put into the regression models and removed if non-significant. Regression models were estimated using ordinary least squares. This was done instead of using a fixed effects model as there were no significant changes in the prediction variables from time one to time two. Variance Inflation Factor analysis was run to inspect multicollinearity, VIF for all variables was below 2 indicating that there was no multicollinearity. Predictor variables age (in all regressions) and Time 1 EF (in regressions that analyse EF change) were entered into the regression first, to evaluate the amount of residual variance predicted by hypothesised, followed by additional protective and risk factor variables. The final additional variables included in the regressions were attending ECCE, diversity of caregivers, books and toys in the home, and family relationships.

## Results

### Descriptive statistics

Tables 2 and 3 present the sample characteristics and descriptive statistics for all analytic variables. Twenty-four percent of caregivers in the current sample completed high school (12 years of school). Seventy-four percent of caregivers reported their income as under R3000/month (~192 USD). The average number of people per household was five. For comparison, South Africa’s food poverty line for 2021 was R624 per person (Hossain et al., 2021; Munambah et al., 2021), i.e., the minimum income an individual needs to meet their daily energy intake), which places even those with a monthly household income of R3000 below the food poverty line.

**Table 2:** Descriptive characteristics of the sample.


CHARACTERISTICS	TIME 1	

Child sex (female)	104 (55%)	

Child attending ECCE services (yes)	T1: 3 (1.6%)	T2: 94 (55%)

Caregiver post school education (yes)	40 (21%)	

Certificate	34 (18%)	

Diploma	5 (3%)	

Degree	1 (0.6%)	

Caregiver relationship to child		

Mother	131 (69%)	

Grandmother	36 (19%)	

Father	9 (5%)	

Aunt	10 (5%)	

Other	3 (2%)	

Monthly household income range		

<R750	16 (9%)	

R750–R1500	52 (28.5%)	

R1500–R3000	70 (38%)	

R3000–R6000	36 (20%)	

R6000–R11000	7 (4%)	

R11000–R27000	0	

>R27000	1 (0.5%)	


*Note:* ECCE = Early childhood care and education.

**Table 3:** Descriptive statistics for continuous variables included in analysis.


	TIME 1				TIME 2			
	
	MEAN	SD	MIN	MAX	MEAN	SD	MIN	MAX

*Executive function*								

Composite score	0	0.72	–1.8	1.78	0	0.71	–2.4	1.18

Inhibition	0.60	0.23	0	1	0.72	0.21	0.10	1

Cognitive flexibility	7.77	2.15	2	12	8.16	2.73	0	12

Working memory	1.54	0.80	0	3.33	1.99	.82	0	4

*Demographics*								

Child age (months)	57	6.9	35.15	70.73	64.2	6.84	42.51	78.61

# languages spoken at home	1.21	0.46	1	3				

Caregiver education	10.15	2.28	1	15				

# children in house	2.98	1.46	1	9				

Household asset score	7.96	3	1	19				

*Home learning environment*								

HLA frequency score	10.34	3.02	3	16				

Diversity of caregivers	1.02	0.43	0	3.25				

Books and toys in home	2.59	0.96	0	4				

Time with child	4.05	1.77	2	6				

*PAFAS*								

Caregiver-child relationship	2.48	2.38	0	13				

Family relationships	3.67	2.51	0	11				

*CECV*								

CECV total score	13.38	10.76	0	59				


*Note:* HLA = home learning activities. PAFAS = Parent and family adjustment scale. CECV = child exposure to community violence.

### Linear regressions

Table 4 presents the linear regression results for the longitudinal prediction of EF at time two. Step 1 (child age predicting EF) was significant, accounting for 21% of time two EF variance. The addition of household and community predictors accounted for significant additional variance (12%, *R^2^ =* 0.33). Significant predictors for EF at time two included age (β = 0.35, *p <* 0.001) and attending to ECCE services (β = 0.30, *p <* 0.001), diversity of caregivers (β = 0.14, *p* = 0.041), and books and toys in the house (β = –0.18, *p* = 0.007).

**Table 4:** Linear regression predicting EF at time 2 (*N* = 171).


*STEP 1*	β	*p* VALUE

Child age	0.29	<0.001

EF at time 1	0.37	<0.001

	*r^2^ = 0.32, p < 0.001*	

***STEP 2***	**β**	***p* VALUE**

Child age	0.22	0.003

EF at time 1	0.32	<0.001

Child attending ECCE services	0.26	<0.001

Diversity of caregivers	0.15	0.022

Books and toys in the house	–0.14	0.031

Family relationships	0.01	0.823

	*r^2^ = 0.41, p < 0.001*	


*Note:* ECCE = early childhood care and education.

Table 5 presents the linear regression results for predictors of residualized change in EF from time one to time two. Step 1 (child age + EF at time 1) was significant, accounting for 32% of the variance. Step 2 (EF at time 1 + household and community factors) was also significant, accounting for an additional 9% of the variance (*R^2^ =* 0.41). Significant predictors for change in EF were the same as Model 1, plus EF at time 1 (β = 0.37, *p <* 0.001): child age (β = 0.22, *p <* 0.001), child attending ECCE services (β = 0.26, *p <* 0.001), diversity of caregivers (β = 0.15, *p* = 0.022), and books and toys in the house (β = –0.14, *p* = 0.031).

**Table 5:** Linear regressions predicting change in EF between Time 1 and Time 2.


*STEP 1*	β	*p* VALUE

Child age	0.35	<0.001

	*r^2^ = 0.21, p < 0.001*	

***STEP 2***	**β**	***p* VALUE**

Child age	0.35	0.001

Child attending ECCE services	0.30	0.001

Diversity of caregivers	0.14	0.041

Books and toys in the house	–0.18	0.007

Family relationships	–0.03	0.642

	*r^2^ = 0.33, p < 0.001*	


*Note:* ECCE = early childhood care and education. *p < 0.05.

## Discussion

The current study aimed to investigate longitudinal risk and protective factors for EF in preschool-age children from low-income settings in South Africa. A child’s age, and if they were attending ECCE services, were the strongest predictors of EF. Diversity of caregivers –the number of caregivers that did learning activities in the home with the child – was the only other household and community factor that was positively associated with EF. Books and toys in the house were also associated with EF but in the reverse direction to what was predicted. These predictors remained the same when looking at change in EF. These results add to accumulating evidence predictors of EF established in Minority World contexts may not be consistent across contexts (Milosavljevic et al., 2023). For example, caregiver education and household socioeconomic status, commonly reported as predictors of EF, were not significant in this sample whereas diversity of caregivers, something that is not often considered in samples from Minority World countries, was significantly associated.

Although this study was aimed at measuring predictors of EF in children that did not have access to ECCE services, due to delays caused by COVID-19 and lockdowns, some children had started their reception year (Grade R), preschool, or creche before time two of data collection. While the amount of exposure to ECCE would have varied within those who commenced (the specific ‘dose’ of which was not captured in this study), merely enrolment in ECCE predicted status and change of EF in this sample. This finding aligns with a study in Zambia that showed children participating in ECCE outperformed their no-ECCE peers on a pencil tap inhibition task by 2.82 times (McCoy et al., 2017). Although in this study we model the prediction of EF by attending ECCE, it is possible that this relationship might work in the opposite direction as well. A study in Zambia showed that children who had better EF were more likely to be enrolled in school early (McCoy, Zuilkowski, et al., 2015), perhaps related to caregiver decisions based on early EF skills predicting a better transition to school (Helm et al., 2020). This is one of the few replications of findings from Minority World settings, such that ECCE services are beneficial for aspects related school readiness and success (Davies et al., 2021; Little, 2021; Sasser et al., 2017).

A recent qualitative study conducted with a sample of caregivers from the same settings as this study revealed that children who were not attending ECCE services tended to have autonomy over their day, whether that was playing outside or watching television (Draper et al., 2023). This is different to children who were attending ECCE services, in which a large portion of their day followed some level of routine and structure. Therefore, it is possible that in this sample, the transition from being at home to a classroom environment might challenge and develop different EF skills to what might be developed in the home and community environment. For instance, EF supports learning by allowing children to work with information in mind, remain focused and resist task-irrelevant distractions, and flexibly shift attention between information and requirements. It is worth noting that the children in the current study were receiving structured activities and stimulation through the home visiting programme. However, the home visits were only once per week for up to 45 minutes at a time, delivered individually, and were aimed at helping the caregiver provide educational stimulation for their child.

The only additional significant household or community predictors were diversity of caregivers, and books and toys in the house. The finding that diversity of caregivers positively predicts EF is in contrast to the null associations found in the recent cross-sectional study in The Gambia and South Africa (Milosavljevic et al., 2023). Diversity of caregivers has not typically been considered in previous EF studies, as most of the research settings in Minority World Countries assume nuclear family structures. More recently, certain aspects of collectivist cultures have been viewed as a strength or ‘hidden talent’ of many Majority World Countries (Ellis et al., 2022), with research starting to consider different family and child care structures (Weber et al., 2021). Multi-generational households are more common in Majority World Countries in which care of children is shared amongst the family and/or community members. In the current study for example, the average number of adults in the house was three, with many caregivers reported living near their extended family who also played a role in caring for their child. The potential reasons that having a variety of caregivers might be EF promotive or protective is not yet known. However, the qualitative interviews with some of the caregivers revealed the importance of social support from their family and community for their own mental health and for the development of their child (Draper et al., 2023).

The family relationships subscale from the PAFAS questionnaire used in the current study asked about family relationships more directly, yet was not associated with EF in the regression analysis. Perhaps the home learning environment questions (asking who did enrichment activities with the child) was a better indicator of social support by highlighting the adults who were involved in caring for the child. There may be other reasons having a diversity of caregivers is beneficial for EF such as requiring the child to negotiate relationships and remember different sets of rules, or the presence of a wider support network for the child. Nonetheless more qualitative work is needed to better characterise family dynamics in these contexts. This is crucial as Western-derived parenting interventions have been criticised for being insensitive to the local culture (Weber et al., 2021) and future work needs to address this.

Books and toys in the house predicted EF in this sample, in the opposite direction to what was expected. No other aspects of the home learning environment (frequency of enrichment activities, time available to spend with child) were significantly associated with EF. Research on the impact of the home learning environment on early cognition and development has yielded mixed results. For example, the non-association in the current study replicated a previous cross-sectional study that found no associations between numeracy skills and the home learning environment (Merkley et al., 2023) and was in line with a longitudinal study conducted in the United States that found no evidence for longitudinal links between the home learning environment (measured using the HOME inventory) and EF at age 15 (Ahmed et al., 2018). While other studies even within South Africa have identified modest, positive associations between home learning opportunities and EF in five-year-old children (Drago et al., 2020). It is possible that as items relied on caregiver report, that the caregivers may be over- or under-reporting the amount of home learning activities or books and toys and thus confounding the results. Or, in the case of books and toys in the home, having books and toys does not necessarily mean that caregivers are using these for enrichment activities with their child. Furthermore, the HLE questionnaire used in the current study focuses on counts of resources and does not ask about quality of resources which is likely to influence the use and impact. On the other hand, the presence of books and toys in the house might be countered by the possibility that less resources could lead to more creative stretching of the imagination requiring EF for creativity and flexible thinking. While the home learning environment assessment was created for use within the South African low-income context, it is possible that the questionnaire does not capture enough of the nuance needed to detect differences in the home learning environment beyond caregiver report.

The non-associations with other predictor variables (exposure to violence, number of siblings, household income, caregiver-child relationship, and caregiver education) both in the bivariate correlations and regression analysis echo results from previous cross-sectional studies in these settings (Cook et al., 2022; Merkley et al., 2023; Milosavljevic et al., 2023), and ongoing research in Majority World Countries is revealing more inconsistencies when it comes to predictors of EF (Mousavi & Gharibzadeh, 2021). Reasons behind these inconsistencies might be related to cultural and contextual norms in these settings. For example, although 80% of the sample were exposed to at least one type of violence, qualitative interviews revealed that caregivers maintained that their community is safe, or that crime is not a problem, despite being quick to give examples of crimes committed in their community (Cook et al., 2022; Draper et al., 2023). Therefore, violence may be normalised to the extent that it does not exert maladaptive or negative effects on EF, or that the effects may only manifest later in childhood. Or that stress from violence is more subjective than is implied by indices that simply measure exposure. This highlights the complexity of these settings and the need to collect more detailed information that could identify whether a certain factor, for e.g., the presence of a father, is a positive or negative thing. This type of nuance might only be captured through mixed methods that include some form of ethnography or qualitative methods. Similar levels of detail may be needed to detect associations with EF when measuring household socio-economic status as well. In the current study, both household assets and income range were used as an indication of household socio-economic status, but neither were associated with EF. Nevertheless, household assets has been found to be a more stable predictor in settings where household income fluctuates by season (Howe et al., 2012). However, factors such as food insecurity and under-nutrition may have more salient effects on EF, and these may not be captured as accurately in questionnaire measures of socioeconomic status. Regarding caregiver education, it is possible that the current sample did not have enough variation in levels of education to detect an impact on EF, with most having done at least some years of high school and only a fifth of the caregivers reporting post-school education. Therefore the positive effect of higher caregiver education on EF may only be seen when levels vary drastically such as those found when comparing mothers with no school and those with secondary school in a Madagascan study (Fernald et al., 2011).

The life history and hidden talents approach provides potential reasons for the inconsistent results found in the current study and others from low-income settings in Majority World countries. This approach suggests that there is cognitive growth resulting from early life adversity, particularly through harsh environments that include threat and unpredictability (Ellis et al., 2022). The cognitive requirements of these contexts (e.g., vigilance, resisting tempting but risky responses, flexible attentional shifting) may confound or even counteract some of the negative effects of adversity on EF. A recent review on the impacts of early life adversity highlighted the complexity of its impacts on children’s mental health and cognitive functioning (Wade et al., 2022). The review identified three distinct but often related forms of early life adversity including threat, deprivation, and unpredictability. There is evidence that shows deprivation (i.e., neglect), but not threat, is associated with lower EF (Sheridan et al., 2017) while the predictability aspect remains understudied. Therefore, future studies should aim to investigate all three aspects of early life adversity taking into account the potential cognitive advantages as well.

Although the caregiver-child relationship did not emerge as a significant predictor of EF in this study, previous qualitative work in these settings (Draper et al., 2023) highlight the crucial role played by caregivers in early childhood development more generally, along with the evidence emphasising the importance of responsive caregiving as part of nurturing care (Britto et al., 2017). Given the challenges of assessing responsive caregiving across cross-cultural settings, it is possible that the current study did not fully capture the aspects of caregiving that positively influence EF in these settings.

## Strengths and Limitations

EF is a relatively new construct to capture in South African contexts, but encouragingly, evidence from this study and previous research shows strong task properties and data distributions approximating those in other Majority World contexts (Cook et al., 2019; Cook et al., 2023; Howard et al., 2020). The current study included a particularly understudied sample, even within South Africa as the majority of studies with preschool-age children are done within the school setting despite the fact that 30% of South African children aged three-five years do not attend an ECCE service (Hall et al., 2019) In doing so, the study had a particular unique (unintended) finding: that commencing some form of ECCE (starting reception year at a formal school or attending an ECCE centre) had a pronounced effect on EF.

Although the longitudinal nature of this study builds on some of the recent cross-sectional work, the time frame between the two rounds of data collection was shorter than intended (due to the COVID-19 pandemic) and potentially not long enough to truly measure the effects of the child’s environment on EF and changes in EF. Encouragingly, this study provides new insights into EF in South African contexts and provides crucial considerations for future studies such as the need to include ethnographic and qualitative methods to capture the context more accurately reflect local norms and perspectives.

## Conclusion

The current study provided further evidence showing that the predictors of EF in Majority World Countries may not look the same as those in Minority World Countries. This study builds directly on previous cross-sectional studies and provides insight and direction for future longitudinal research. It also highlighted the need to use qualitative and ethnographic methods to collect more detailed information about children’s environments, from the perspective of the child, family, and community to better understand and interpret the potential impact on EF and other aspects of development. The finding that attending ECCE services positively predicted EF, highlights the benefits of ECCE services and provides additional data to support the prioritising and funding of ECCE for children from low-income settings.

## Data Accessibility Statement

In compliance with the requirements of our institutional review board, data sharing would be possible: for replication or analysis aligned to the data use participants consented to; with a data sharing agreement between the 2 institutions. Such requests should be directed to the corresponding author (cayjayde7@gmail.com) and the senior author, (catherine.draper@wits.ac.za) and will be considered by the co-author team.

## Additional File

The additional file for this article can be found as follows:

10.5334/joc.377.s1 Supplementary Material. Table S1: Bivariate correlations between variables of interest.

## References

[1] Ahmed, S., Tang, S., Waters, N., & Davis-Kean, P. (2018). Executive Function and Academic Achievement: Longitudinal Relations From Early Childhood to Adolescence. Journal of Educational Psychology, 111. DOI: 10.1037/edu0000296

[2] Alam, S. (2019). Majority World: Challenging the West’s Rhetoric of Democracy. Amerasia Journal. DOI: 10.17953/amer.34.1.l3176027k4q614v5

[3] Blair, C. (2010). Stress and the Development of Self-Regulation in Context. Child Development Perspectives, 4(3), 181–188. DOI: 10.1111/j.1750-8606.2010.00145.x 21779305 PMC3138186

[4] Blair, C., Raver, C. C., & Berry, D. J. (2014). Two approaches to estimating the effect of parenting on the development of executive function in early childhood. Developmental Psychology, 50, 554–565. DOI: 10.1037/a0033647 23834294 PMC4682354

[5] Blakey, E., Matthews, D., Cragg, L., Buck, J., Cameron, D., Higgins, B., Pepper, L., Ridley, E., Sullivan, E., & Carroll, D. J. (2020). The Role of Executive Functions in Socioeconomic Attainment Gaps: Results From a Randomized Controlled Trial. Child Development, 91(5), 1594–1614. DOI: 10.1111/cdev.13358 32031254

[6] Bradley, R., & Corwyn, R. (2005). Caring for children around the world: A view from HOME. International Journal of Behavioral Development, 29, 468–478. DOI: 10.1177/01650250500146925

[7] Britto, P. R., Lye, S. J., Proulx, K., Yousafzai, A. K., Matthews, S. G., Vaivada, T., Perez-Escamilla, R., Rao, N., Ip, P., Fernald, L. C. H., MacMillan, H., Hanson, M., Wachs, T. D., Yao, H., Yoshikawa, H., Cerezo, A., Leckman, J. F., & Bhutta, Z. A. (2017). Nurturing care: Promoting early childhood development. The Lancet, 389(10064), 91–102. DOI: 10.1016/S0140-6736(16)31390-3 27717615

[8] Camerota, M., Willoughby, M. T., & Blair, C. B. (2020). Measurement models for studying child executive functioning: Questioning the status quo. Developmental Psychology, 56(12), 2236–2245. DOI: 10.1037/dev0001127 33104374 PMC8284867

[9] Cole, K., & Mitchell, P. (2000). Siblings in the development of executive control and a theory of mind. British Journal of Developmental Psychology, 18(2), 279–295. DOI: 10.1348/026151000165698

[10] Cook, C., Howard, S., Scerif, G., Twine, R., Kahn, K., Norris, S., & Draper, C. (2019). Associations of physical activity and gross motor skills with executive function in preschool children from low-income South African settings. Developmental Science, 22(5), e12820. DOI: 10.1111/desc.12820 30801916

[11] Cook, C. J., Howard, S. J., Cuartas, J., Makaula, H., Merkley, R., Mshudulu, M., Tshetu, N., Scerif, G., & Draper, C. E. (2022). Child exposure to violence and self-regulation in South African preschool-age children from low-income settings. Child Abuse & Neglect, 134, 105944. DOI: 10.1016/j.chiabu.2022.105944 36356426

[12] Cook, C. J., Howard, S., Scerif, G., Twine, R., Kahn, K., Norris, S., & Draper, C. (2023). Executive function and pre-academic skills in preschoolers from South Africa. South African Journal of Childhood Education, 13(1), Article 1. DOI: 10.4102/sajce.v13i1.1369

[13] Davies, C., Hendry, A., Gibson, S. P., Gliga, T., McGillion, M., & Gonzalez-Gomez, N. (2021). Early childhood education and care (ECEC) during COVID-19 boosts growth in language and executive function. Infant and Child Development, 30(4), e2241. DOI: 10.1002/icd.2241 34220356 PMC8236989

[14] Dawes, A., Biersteker, L., Girdwood, L., & Snelling, M. (2020). The Early Learning Programme Outcomes Study Technical Report.

[15] Doebel, S. (2020). Rethinking executive function and its development. Perspectives on Psychological Science, 15(4), 942–956. DOI: 10.1177/1745691620904771 32348707

[16] Donald, K. A., Hoogenhout, M., du Plooy, C. P., Wedderburn, C. J., Nhapi, R. T., Barnett, W., Hoffman, N., Malcolm-Smith, S., Zar, H. J., & Stein, D. J. (2018). Drakenstein Child Health Study (DCHS): Investigating determinants of early child development and cognition. BMJ Paediatrics Open, 2(1), e000282. DOI: 10.1136/bmjpo-2018-000282 29942867 PMC6014194

[17] Drago, F., Scharf, R. J., Maphula, A., Nyathi, E., Mahopo, T. C., Svensen, E., Mduma, E., Bessong, P., & Rogawski McQuade, E. T. (2020). Psychosocial and environmental determinants of child cognitive development in rural south africa and tanzania: Findings from the mal-ed cohort. BMC Public Health, 20(1), 505. DOI: 10.1186/s12889-020-08598-5 32299410 PMC7164138

[18] Draper, C. E., Cook, C. J., Howard, S. J., Makaula, H., Merkley, R., Mshudulu, M., … & Scerif, G. (2023). Caregiver perspectives of risk and protective factors influencing early childhood development in low-income, urban settings: A social ecological perspective. Infant and Child Development, 32(3), e2417. DOI: 10.1002/icd.2417

[19] Draper, C. E., Achmat, M., Forbes, J., & Lambert, E. V. (2012). Impact of a community-based programme for motor development on gross motor skills and cognitive function in preschool children from disadvantaged settings from disadvantaged settings. Early Child Development and Care, 182(1), 137–152. DOI: 10.1080/03004430.2010.547250

[20] Draper, C. E., Barnett, L. M., Cook, C. J., Cuartas, J. A., Howard, S. J., McCoy, D. C., Merkley, R., Molano, A., Maldonado-Carreño, C., Obradović, J., Scerif, G., Valentini, N. C., Venetsanou, F., & Yousafzai, A. K. (2022). Publishing child development research from around the world: An unfair playing field resulting in most of the world’s child population underrepresented in research. Infant and Child Development.

[21] Draper, C., Tomaz, S. A., Cook, C. J., Jugdav, S. S., Ramsammy, C., Besharati, S., Van Heerden, A., Vilakazi, K., Cockcroft, K., Howard, S. J., & Okely, A. D. (2020). Understanding the influence of 24-hour movement behaviours on the health and development of preschool children from low-income South African settings: The SUNRISE pilot study. South African Journal of Sports Medicine, 32(1), 1–7. DOI: 10.17159/2078-516X/2020/v32i1a8415 PMC9924532 36818976

[22] Duran, C. A. K., Cottone, E., Ruzek, E. A., Mashburn, A. J., & Grissmer, D. W. (2020). Family Stress Processes and Children’s Self-Regulation. Child Development, 91(2), 577–595. DOI: 10.1111/cdev.13202 30585628

[23] Ellis, B. J., Abrams, L. S., Masten, A. S., Sternberg, R. J., Tottenham, N., & Frankenhuis, W. E. (2022). Hidden talents in harsh environments. Development and Psychopathology, 34(1), 95–113. DOI: 10.1017/S0954579420000887 32672144

[24] Evans, G. W., & Kim, P. (2013). Childhood Poverty, Chronic Stress, Self-Regulation, and Coping. Child Development Perspectives, 7(1), 43–48. DOI: 10.1111/cdep.12013

[25] Fernald, L., Weber, A., Galasso, E., & Ratsifandrihamanana, L. (2011). Socioeconomic gradients and child development in a very low income population: Evidence from Madagascar. Developmental Science, 14, 832–847. DOI: 10.1111/j.1467-7687.2010.01032.x 21676102

[26] Fuhs, M., Nesbitt, K., Farran, D., & Dong, N. (2014). Longitudinal Associations Between Executive Functioning and Academic Skills Across Content Areas. Developmental Psychology, 50. DOI: 10.1037/a0036633 24749550

[27] Ganzel, B. L., Morris, P. A., & Wethington, E. (2010). Allostasis and the human brain: Integrating models of stress from the social and life sciences. Psychological Review, 117(1), 134–174. DOI: 10.1037/a0017773 20063966 PMC2808193

[28] Hackman, D. A., Gallop, R., Evans, G. W., & Farah, M. J. (2015). Socioeconomic status and executive function: Developmental trajectories and mediation. Developmental Science, 18(5), 686–702. DOI: 10.1111/desc.12246 25659838

[29] Haft, S. L., & Hoeft, F. (2017). Poverty’s Impact on Children’s Executive Functions: Global Considerations. New Directions for Child and Adolescent Development, 2017(158), 69–79. DOI: 10.1002/cad.20220 29243384 PMC5913739

[30] Halgunseth, L. C., Ispa, J. M., & Rudy, D. (2006). Parental Control in Latino Families: An Integrated Review of the Literature. Child Development, 77(5), 1282–1297. DOI: 10.1111/j.1467-8624.2006.00934.x 16999798

[31] Hall, K., Sambu, W., Almeleh, C., Mabaso, C., Giese, S., & Proudlock, P. (2019). South Africa Early Childhood Review 2019. Children’s Institute, University of Cape Town and Ilifa Labantwana.

[32] Hamilton, L. G., Hayiou-Thomas, M. E., Hulme, C., & Snowling, M. J. (2016). The Home Literacy Environment as a Predictor of the Early Literacy Development of Children at Family-Risk of Dyslexia. Scientific Studies of Reading, 20(5), 401–419. DOI: 10.1080/10888438.2016.1213266 28250707 PMC5308453

[33] Haslam, D., Mejia, A., Thomson, D., & Betancourt, T. (2019). Self-Regulation in Low- and Middle-Income Countries: Challenges and Future Directions. Clinical Child and Family Psychology Review, 22. DOI: 10.1007/s10567-019-00278-0 30725308

[34] Helm, A. F., McCormick, S. A., Deater-Deckard, K., Smith, C. L., Calkins, S. D., & Bell, M. A. (2020). Parenting and Children’s Executive Function Stability Across the Transition to School. Infant and Child Development, 29(1), e2171. DOI: 10.1002/icd.2171 32617081 PMC7331947

[35] Henrich, J., Heine, S. J., & Norenzayan, A. (2010). The weirdest people in the world? Behavioral and Brain Sciences, 33(2–3), 61–83. DOI: 10.1017/S0140525X0999152X 20550733

[36] Hossain, M. S., Deeba, I. M., Hasan, M., Kariippanon, K. E., Chong, K. H., Cross, P. L., Ferdous, S., & Okely, A. D. (2021). International study of 24-h movement behaviors of early years (SUNRISE): A pilot study from Bangladesh. Pilot and Feasibility Studies, 7(1), 176. DOI: 10.1186/s40814-021-00912-1 34526148 PMC8440144

[37] Howard, S. J., Cook, C. J., Everts, L., Melhuish, E., Scerif, G., Norris, S., Twine, R., Kahn, K., & Draper, C. E. (2020). Challenging socioeconomic status: A cross-cultural comparison of early executive function. Developmental Science, 23(1), e12854. DOI: 10.1111/desc.12854 31077525

[38] Howard, S. J., & Melhuish, E. (2017). An Early Years Toolbox for Assessing Early Executive Function, Language, Self-Regulation, and Social Development: Validity, Reliability, and Preliminary Norms. Journal of Psychoeducational Assessment, 35(3), 255–275. DOI: 10.1177/0734282916633009 28503022 PMC5424850

[39] Howe, L. D., Galobardes, B., Matijasevich, A., Gordon, D., Johnston, D., Onwujekwe, O., Patel, R., Webb, E. A., Lawlor, D. A., & Hargreaves, J. R. (2012). Measuring socio-economic position for epidemiological studies in low- and middle-income countries: A methods of measurement in epidemiology paper. International Journal of Epidemiology, 41(3), 871–886. DOI: 10.1093/ije/dys037 22438428 PMC3396323

[40] Hughes, C., & Devine, R. T. (2019). For Better or for Worse? Positive and Negative Parental Influences on Young Children’s Executive Function. Child Development, 90(2), 593–609. DOI: 10.1111/cdev.12915 28800148

[41] Jasińska, K., Zinszer, B., Xu, Z., Hannon, J., Seri, A. B., Tanoh, F., & Akpé, H. (2022). Home Learning Environment and Physical Development Impact Children’s Executive Functions and Literacy in Rural Côte d’Ivoire. Cognitive Development, 64(101265). DOI: 10.1016/j.cogdev.2022.101265

[42] Khan, T., Abimbola, S., Kyobutungi, C., & Pai, M. (2022). How we classify countries and people—And why it matters. BMJ Global Health, 7(6), e009704. DOI: 10.1136/bmjgh-2022-009704 PMC9185389 35672117

[43] Lawson, G. M., & Farah, M. J. (2017). Executive function as a mediator between SES and academic achievement throughout childhood. International Journal of Behavioral Development, 41(1), 94–104. DOI: 10.1177/0165025415603489 28082756 PMC5222613

[44] Lessing, A. C., & De Witte, M. W. (2005). An investigation into the early literacy skills of Grade R second-language (L2) learners in South Africa. Africa Education Review, 2(2), 242–257. DOI: 10.1080/18146620508566303

[45] Little, M. (2021). Nationally Representative Evidence on the Association Between Preschool and Executive Function Skills Throughout Elementary School. AERA Open, 7, 23328584211048399. DOI: 10.1177/23328584211048399

[46] Lohndorf, R., Vermeer, H. J., la Harpe, C., & Mesman, J. (2021). Socioeconomic status, parental beliefs, and parenting practices as predictors of preschoolers’ school readiness and executive functions in chile. Early Childhood Research Quarterly, 57, 61–74. DOI: 10.1016/j.ecresq.2021.05.001

[47] Martin, L., Revington, N., & Seedat, S. (2013). The 39-Item Child Exposure to Community Violence (CECV) Scale: Exploratory Factor Analysis and Relationship to PTSD Symptomatology in Trauma-Exposed Children and Adolescents. International Journal of Behavioral Medicine, 20(4), 599–608. DOI: 10.1007/s12529-012-9269-7 23055027

[48] McAlister, A., & Peterson, C. C. (2006). Mental playmates: Siblings, executive functioning and theory of mind. British Journal of Developmental Psychology, 24(4), 733–751. DOI: 10.1348/026151005X70094

[49] McCoy, D. C. (2013). Early Violence Exposure and Self-Regulatory Development: A Bioecological Systems Perspective. Human Development, 56(4), 254–273. DOI: 10.1159/000353217

[50] McCoy, D. C., Raver, C. C., & Sharkey, P. (2015). Children’s Cognitive Performance and Selective Attention Following Recent Community Violence. Journal of Health and Social Behavior, 56(1), 19–36. DOI: 10.1177/0022146514567576 25663176 PMC4671628

[51] McCoy, D. C., Roy, A. L., & Raver, C. C. (2016). Neighborhood crime as a predictor of individual differences in emotional processing and regulation. Developmental Science, 19(1), 164–174. DOI: 10.1111/desc.12287 25702532 PMC5111804

[52] McCoy, D. C., Zuilkowski, S. S., & Fink, G. (2015). Poverty, physical stature, and cognitive skills: Mechanisms underlying children’s school enrollment in Zambia. Developmental Psychology, 51(5), 600–614. DOI: 10.1037/a0038924 25844851

[53] McCoy, D. C., Zuilkowski, S. S., Yoshikawa, H., & Fink, G. (2017). Early Childhood Care and Education and School Readiness in Zambia. Journal of Research on Educational Effectiveness, 10(3), 482–506. DOI: 10.1080/19345747.2016.1250850

[54] Melhuish, E. (2010). Impact of the home learning environment on child cognitive development: Secondary analysis of the data from ‘Growing up in Scotland’. Scottish Government Social Research.

[55] Merkley, R., Sernoskie, E., Cook, C. J., Howard, S. J., Makaula, H., Mshudulu, M., Tshetu, N., Draper, C. E., & Scerif, G. (2023). ‘We don’t have things for counting’: An exploration of early numeracy skills and home learning experiences of children growing up in poverty in South Africa. Journal of Numerical Cognition. https://www.psycharchives.org/en/item/12a6ac0e-e787-49ec-9756-fb4f8ce6848a. DOI: 10.5964/jnc.8061

[56] Metaferia, B. K., Futo, J., & Takacs, Z. K. (2021). Parents’ Views on Play and the Goal of Early Childhood Education in Relation to Children’s Home Activity and Executive Functions: A Cross-Cultural Investigation. Frontiers in Psychology, 12. https://www.frontiersin.org/articles/10.3389/fpsyg.2021.646074. DOI: 10.3389/fpsyg.2021.646074 PMC8108989 33981273

[57] Milosavljevic, B., Cook, C. J., Fadera, T., Ghillia, G., Howard, S. J., Makaula, H., Mbye, E., McCann, S., Merkley, R., Mshudulu, M., Saidykhan, M., Touray, E., Tshetu, N., Elwell, C., Moore, S. E., Scerif, G., Draper, C. E., & Lloyd-Fox, S. (2023). Executive functioning skills and their environmental predictors among pre-school aged children in South Africa and The Gambia. Developmental Science, e13407. DOI: 10.1111/desc.13407 37128134

[58] Miyake, A., & Friedman, N. P. (2012). The Nature and Organization of Individual Differences in Executive Functions: Four General Conclusions. Current Directions in Psychological Science, 21(1), 8–14. DOI: 10.1177/0963721411429458 22773897 PMC3388901

[59] Moffitt, T. E., Arseneault, L., Belsky, D., Dickson, N., Hancox, R. J., Harrington, H., Houts, R., Poulton, R., Roberts, B. W., Ross, S., Sears, M. R., Thomson, W. M., & Caspi, A. (2011). A gradient of childhood self-control predicts health, wealth, and public safety. Proceedings of the National Academy of Sciences, 108(7), 2693–2698. DOI: 10.1073/pnas.1010076108 PMC3041102 21262822

[60] Morelli, G., Quinn, N., Chaudhary, N., Vicedo, M., Rosabal-Coto, M., Keller, H., Murray, M., Gottlieb, A., Scheidecker, G., & Takada, A. (2018). Ethical Challenges of Parenting Interventions in Low- to Middle-Income Countries. Journal of Cross-Cultural Psychology, 49(1), 5–24. DOI: 10.1177/0022022117746241

[61] Mousavi, S. Z., & Gharibzadeh, S. (2021). Growing up in a challenging environment: A cultural analysis of self-regulation development in poverty. European Journal of Developmental Psychology, 1–18. DOI: 10.1080/17405629.2021.1928490

[62] Munambah, N., Gretschel, P., Muchirahondo, F. C., Chiwaridzo, M., Chikwanha, T. M., Kariippanon, K. E., Chong, K. H., Cross, P. L., Draper, C. E., & Okely, A. D. (2021). 24 hour movement behaviours and the health and development of pre-school children from Zimbabwean settings: The SUNRISE pilot study. South African Journal of Sports Medicine, 33(1). DOI: 10.17159/2078-516X/2021/v33i1a10864 PMC9924604 36816901

[63] Naudé, H., Pretorius, E., & Viljoen, J. (2003). The Impact of Impoverished Language Development on Preschoolers’ Readiness-To-Learn During the Foundation Phase. Early Child Development and Care, 173(2–3), 271–291. DOI: 10.1080/03004430303098

[64] Nielsen, M., Haun, D., Kärtner, J., & Legare, C. H. (2017). The persistent sampling bias in developmental psychology: A call to action. Journal of Experimental Child Psychology, 162, 31. DOI: 10.1016/j.jecp.2017.04.017 28575664 PMC10675994

[65] Nweze, T., Nwoke, M. B., Nwufo, J. I., Aniekwu, R. I., & Lange, F. (2021). Working for the future: Parentally deprived Nigerian Children have enhanced working memory ability. Journal of Child Psychology and Psychiatry, 62(3), 280–288. DOI: 10.1111/jcpp.13241 32302431

[66] Obradović, J., Portilla, X. A., Tirado-Strayer, N., Siyal, S., Rasheed, M. A., & Yousafzai, A. K. (2017). Maternal scaffolding in a disadvantaged global context: The influence of working memory and cognitive capacities. Journal of Family Psychology, 31(2), 139–149. DOI: 10.1037/fam0000279 28068110

[67] Obradović, J., & Willoughby, M. T. (2019). Studying Executive Function Skills in Young Children in Low- and Middle-Income Countries: Progress and Directions. Child Development Perspectives, 13(4), 227–234. DOI: 10.1111/cdep.12349

[68] Rathore, M. A., Armstrong-Carter, E., Siyal, S., Yousafzai, A. K., & Obradović, J. (2022). Pakistani preschoolers’ number of older siblings and cognitive skills: Moderations by home stimulation and gender. Journal of Family Psychology, 132–142. DOI: 10.1037/fam0001018 35913843

[69] Raver, C. C., Blair, C., & Willoughby, M. (2013). Poverty as a Predictor of 4-Year-Olds’ Executive Function: New Perspectives on Models of Differential Susceptibility. Developmental Psychology, 49(2), 292–304. DOI: 10.1037/a0028343 22563675 PMC5460626

[70] Richter, L., & Samuels, M-L. (2018). The South African universal preschool year: A case study of policy development and implementation. Child: Care, Health and Development, 44(1), 12–18. DOI: 10.1111/cch.12511 29235166

[71] Röthlisberger, M., Neuenschwander, R., Michel, E., & Roebers, C. (2010). Exekutive Funktionen: Zugrundeliegende kognitive Prozesse und deren Korrelate bei Kindern im späten Vorschulalter. Zeitschrift Fur Entwicklungspsychologie Und Padagogische Psychologie – Z ENTWICKLUNGSPSYCHOL PADAGO, 42, 99–110. DOI: 10.1026/0049-8637/a000010

[72] Sanders, M. R., Morawska, A., Haslam, D. M., Filus, A., & Fletcher, R. (2014). Parenting and Family Adjustment Scales (PAFAS): Validation of a Brief Parent-Report Measure for Use in Assessment of Parenting Skills and Family Relationships. Child Psychiatry & Human Development, 45(3), 255–272. DOI: 10.1007/s10578-013-0397-3 23955254

[73] Sasser, T. R., Bierman, K. L., Heinrichs, B., & Nix, R. L. (2017). Preschool Intervention Can Promote Sustained Growth in the Executive-Function Skills of Children Exhibiting Early Deficits. Psychological Science, 28(12), 1719–1730. DOI: 10.1177/0956797617711640 29065281 PMC5725267

[74] Save the Children. (2016). IDELA Early Childhood Pilot Study in South Africa. Save the Children. https://idela-network.org/data-set/early-childhood-pilot-study-using-idela-south-africa/

[75] Sheridan, M. A., Peverill M, M., Finn, A. S., & Mclaughlin, K. A. (2017). Dimensions of childhood adversity have distinct associations with neural systems underlying executive functioning. Development and Psychopathology, 29(5), 1777–1794. DOI: 10.1017/S0954579417001390 29162183 PMC5733141

[76] Snelling, M., & Dawes, A. (2010). The Innovation Edge Home Learning Environment Tool Psychometry (p. 10).

[77] StataCorp. (2021). Stata Statistical Software: Release 17. College Station, TX: StataCorp LLC.

[78] Statistics South Africa. (2011). Census 2011.

[79] Suntheimer, N. M., Wolf, S., Sulik, M. J., Avornyo, E. A., & Obradović, J. (2022). Executive function mediates the association between cumulative risk and learning in Ghanaian schoolchildren. Developmental Psychology, 58(8), 1500–1511. DOI: 10.1037/dev0001372 35446075

[80] Tredoux, C., Dawes, A., Mattes, F., Schenk, J.-C., Giese, S., Leach, G., van der Berg, S., & Horler, J. (2024). Are South African children on track for early learning? Findings from the South African Thrive By Five Index 2021 Survey. Child Indicators Research, 17(2), 601–636. DOI: 10.1007/s12187-023-10093-3

[81] Vrantsidis, D. M., Clark, C. A. C., Chevalier, N., Espy, K. A., & Wiebe, S. A. (2020). Socioeconomic status and executive function in early childhood: Exploring proximal mechanisms. Developmental Science, 23(3), e12917. DOI: 10.1111/desc.12917 31680392

[82] Wade, M., Wright, L., & Finegold, K. E. (2022). The effects of early life adversity on children’s mental health and cognitive functioning. Translational Psychiatry, 12(1), 244. DOI: 10.1038/s41398-022-02001-0 35688817 PMC9187770

[83] Weber, A. M., Diop, Y., Gillespie, D., Ratsifandrihamanana, L., & Darmstadt, G. L. (2021). Africa is not a museum: The ethics of encouraging new parenting practices in rural communities in low-income and middle-income countries. BMJ Global Health, 6(7), e006218. DOI: 10.1136/bmjgh-2021-006218 PMC8286753 34266849

[84] Willoughby, M., Piper, B., Oyanga, A., & Merseth, K. (2019). Measuring Executive Function Skills in Young Children in Kenya: Associations with School Readiness. Developmental Science, 22, e12818. DOI: 10.1111/desc.12818 30779264

[85] Wolf, S., & McCoy, D. C. (2019). The role of executive function and social-emotional skills in the development of literacy and numeracy during preschool: A cross-lagged longitudinal study. Developmental Science, 22(4), e12800. DOI: 10.1111/desc.12800 30666761

[86] Zysset, A. E., Kakebeeke, T. H., Messerli-Bürgy, N., Meyer, A. H., Stülb, K., Leeger-Aschmann, C. S., Schmutz, E. A., Arhab, A., Puder, J. J., Kriemler, S., Munsch, S., & Jenni, O. G. (2018). Predictors of Executive Functions in Preschoolers: Findings From the SPLASHY Study. Frontiers in Psychology, 9. https://www.frontiersin.org/articles/10.3389/fpsyg.2018.02060. DOI: 10.3389/fpsyg.2018.02060 PMC6216414 30420823

