# Supplementary material for: Risk and Protective Factors for Executive Function in Vulnerable South African Preschool-Age Children
Source: J Cogn. 2024 Jul 18;7(1):58. doi: 10.5334/joc.377 (PMC11259110; doi:10.5334/joc.377)
Supplement: Supplementary Material. — Table S1: Bivariate correlations between variables of interest. [file joc-7-1-377-s1.pdf]

Table S1: Bivariate correlations between variables of interest

Variables	(1)	(2)	(3)	(4)	(5)	(6)	(7)	(8)	(9)	(10)	(11)	(12)	(13)	(14)	(15)
(1) EF composite score	1.00														
(2) Child age	0.471	1.00													
(3) Child attending ECCE	0.445	0.379	1.00												
(4) Household income range	0.093	0.021	0.080	1.00											
(5) Household asset score	0.116	-0.023	0.233	0.343	1.00										
(6) # children in the house	-0.048	0.085	-0.053	0.206	0.006	1.00									
(7) # languages spoken in home	0.046	0.072	-0.068	-0.018	0.049	0.110	1.00								
(8) Caregiver education	0.025	-0.038	0.205	0.168	0.271	-0.155	-0.071	1.00							
(9) CECV total score	-0.045	0.170	-0.097	-0.006	-0.168	0.017	0.120	-0.115	1.00						
(10) Caregiver-child relationship	-0.103	-0.011	-0.200	-0.237	-0.193	0.126	0.159	-0.228	-0.060	1.00					
(11) Family relationship score	-0.191	-0.144	-0.253	-0.057	-0.175	-0.026	0.111	-0.110	0.197	0.204	1.00				
(12) HLA frequency score	0.068	0.124	0.172	0.088	0.163	0.135	0.214	-0.049	0.075	-0.227	0.087	1.00			
(13) Diversity of caregivers	0.181	0.104	0.110	0.183	0.215	0.102	0.266	0.110	0.064	-0.239	-0.121	0.480	1.00		
(14) Books and toys in home	-0.103	0.072	0.040	0.150	0.207	-0.004	0.140	0.122	0.149	-0.145	-0.046	0.335	0.249	1.00	
(15) Time with child	0.045	0.149	0.146	0.109	0.039	0.004	0.157	-0.014	0.140	-0.389	-0.224	0.239	0.277	0.359	1.00
